# Characterization of Adsorption Enthalpy of Novel Water-Stable Zeolites and Metal-Organic Frameworks

**DOI:** 10.1038/srep19097

**Published:** 2016-01-22

**Authors:** Hyunho Kim, H. Jeremy Cho, Shankar Narayanan, Sungwoo Yang, Hiroyasu Furukawa, Scott Schiffres, Xiansen Li, Yue-Biao Zhang, Juncong Jiang, Omar M. Yaghi, Evelyn N. Wang

**Affiliations:** 1 Department of Mechanical Engineering, Massachusetts Institute of Technology, 77 Massachusetts Ave, Cambridge, MA 02139, United States; 2 Department of Chemistry, University of California – Berkeley, Materials Sciences Division, Lawrence Berkeley National Laboratory, and Kavli Energy NanoSciences Institute at Berkeley, Berkeley, California 94720, United States

## Abstract

Water adsorption is becoming increasingly important for many applications including thermal energy storage, desalination, and water harvesting. To develop such applications, it is essential to understand both adsorbent-adsorbate and adsorbate-adsorbate interactions, and also the energy required for adsorption/desorption processes of porous material-adsorbate systems, such as zeolites and metal-organic frameworks (MOFs). In this study, we present a technique to characterize the enthalpy of adsorption/desorption of zeolites and MOF-801 with water as an adsorbate by conducting desorption experiments with conventional differential scanning calorimetry (DSC) and thermogravimetric analyzer (TGA). With this method, the enthalpies of adsorption of previously uncharacterized adsorbents were estimated as a function of both uptake and temperature. Our characterizations indicate that the adsorption enthalpies of type I zeolites can increase to greater than twice the latent heat whereas adsorption enthalpies of MOF-801 are nearly constant for a wide range of vapor uptakes.

Estimation of the adsorption enthalpy is essential for many applications including adsorption heating and cooling^1^,^2^,^3^,^4^,^5^,^6^,^7^,^8^,^9^, adsorption desalination^10^,^11^,^12^, and gas separation and storage systems with adsorbents^13^,^14^,^15^. The adsorption enthalpy is an important parameter for modeling such systems efficiently because it dictates the energy required to operate, or the energy densities of, these systems. Due to the high enthalpy of adsorption and evaporation/condensation, and its zero global warming potential, various adsorbent-water systems have received significant attention for adsorption heating and cooling applications^1^,^3^,^4^,^5^,^6^, as the average enthalpy of adsorption is typically higher than the latent heat of evaporation^16^. In addition, water capture by adsorption at low relative humidity can deliver fresh water without the use of electric power^17^,^18^. The average enthalpy of adsorption is found to increase with adsorbents with IUPAC type I behavior compared to other types^16^,^19^, with higher affinity to water molecules. The most studied hydrophilic adsorbents are zeolites, but recently developed metal-organic frameworks (MOFs) also have strong hydrophilic properties with stable cyclic hydrothermal performance^17^,^20^,^21^,^22^,^23^.

The enthalpy of adsorption is most commonly estimated in either of the two following ways: estimation of the isosteric enthalpy of adsorption and direct calorimetric measurements with the use of the Tian-Calvet calorimeter^19^. The isosteric enthalpy of adsorption is a thermodynamic relation derived from the adsorption equilibrium measured at different temperatures with constant uptake, also known as the Clausius-Clapeyron relation, given by


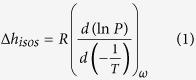


where 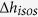, 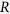, 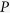, 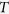, and 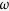 represent the isosteric enthalpy of adsorption, universal gas constant, pressure, temperature, and vapor uptake, respectively. The isosteric enthalpy of adsorption is obtained as a function of the uptake by using Eqn (1) and adsorption isotherms measured across a wide temperature range, as shown in Fig. 1 (a). This was carried out for the MOF-801^17^ and water pair with a linear interpolation method, as shown in Fig. 1 (b). To use Eqn (1), we must assume ideal gas behavior in the gaseous phase, negligible volume of the adsorbed species in comparison to the gaseous phase, reversible physisorption, inertness of the adsorbent, and that thermodynamic equilibrium was reached. Adsorbents used in this study are considered to be physisorbents^9^,^17^,^24^. Since previous studies have also shown good agreement between the isosteric method and calorimetric measurements, for N_2_ and O_2_ adsorbates with zeolite CaA, and CO_2_ adsorbate with zeolite 13X pairs^25^, the isosteric method is often used to characterize the differential energy for the adsorption process^17^,^20^,^26^. However, as with many hydrophilic adsorbents, the separation between different temperature isotherms at low relative pressures (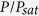, absolute pressure over saturation pressure) is minimal, making them challenging to discern due to experimental resolution and uncertainty limitations (see Fig. 1 (a) for 13X and MgY zeolites). As such, vapor adsorption capacity obtained for 13X and MgY zeolites were 24.6wt.% and 28.4wt.% at 1% relative pressure at 25 °C, an absolute pressure near 30 Pa, shown in Fig. 1 (a), respectively. Consequently, the isosteric method is highly sensitive to the resolution of adsorption isotherms and the interpolation techniques^27^, making calorimetric methods more suitable. However, the calorimetric method to measure the differential and the integral enthalpy of adsorption (the latter using an average enthalpy between a state 1 to 2^19^) requires specialized equipment^19^,^28^, which is not widely available in academic and industrial facilities. In this paper, we present a new experimental technique and thermodynamic model using conventional differential scanning calorimetry (DSC) and thermogravimetric analyzer (TGA) systems that can be used to characterize the enthalpy of adsorption. With this method, we obtained the enthalpy of adsorption of water vapor for novel adsorbents, such as MgY zeolite^24^ and MOF-801^17^, which can be used in a wide range of applications, including thermal energy storage, climate control, and water purification.

## Results

### DSC and TGA experiments

**Figure 1:**
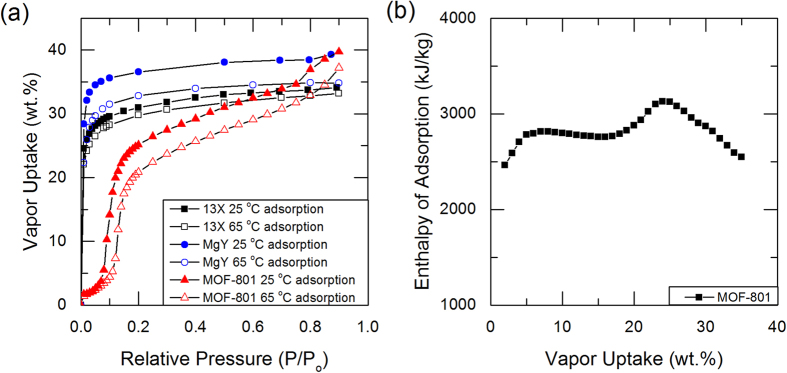
Adsorption isotherms and isosteric enthalpy of adsorption. (**a**) Adsorption isotherms (vapor uptake in weight percent vs. relative pressure, absolute pressure normalized by saturation pressure) of 13X and MgY zeolites, and MOF-801 with water pairs characterized with dynamic vapor sorption analyzer (DVS Vacuum, Surface Measurement Systems Ltd., London, UK). Adsorbents were regenerated with high vacuum (<1Pa) with a temperature greater than 100 °C (**b**) Isosteric enthalpy of adsorption calculated using Eqn (1) and isotherms shown in (**a**) for MOF-801 and water pair with the linear interpolation method.

**Figure 2:**
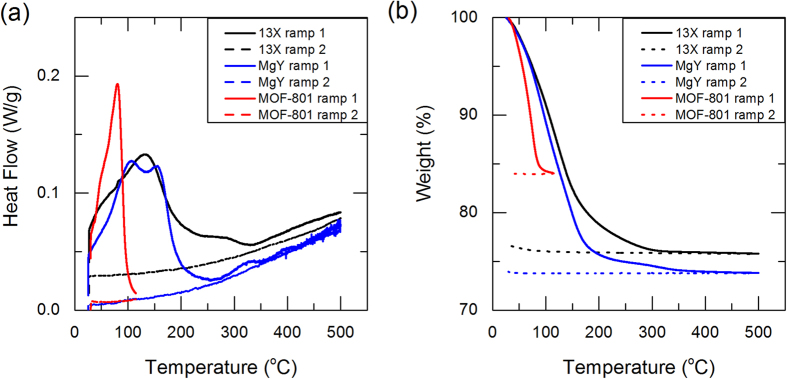
(a) DSC and (b) TGA results of 13X and MgY zeolites, and MOF-801 with water pairs. Data shown in this plot is obtained with 1 °C/min temperature ramp. Weights of saturated samples used in DSC experiments are 5.84 mg, 4.56 mg, and 9.37 mg for 13X, MgY, and MOF-801, respectively. Magnitude of heat flow is not important in DSC measurements as only relative heat flow between ramps 1 and 2 is considered in the calculation.

The adsorbents used in this study are 13X (molecular sieves 13X, powder, ~2 μm avg. part. size, Sigma Aldrich) and MgY^24^ zeolites, and recently reported MOF-801^17^. Partially saturated adsorbents at 60% relative humidity produced by mixture of nitrogen gas and deionized water vapor were prepared in a vapor sorption analyzer (Q5000SA, TA Instruments), and tested in a DSC (Discovery DSC, TA Instruments) and a TGA (Discovery TGA, TA Instruments) with various temperature ramp rates. Zeolite samples were heated up to 500 °C and MOF-801 samples were heated up to 115 °C, and temperature ramps were repeated twice during each experiments: the first for desorption heat transfer and the second for sensible heat transfer. Detailed sample preparation and experimental procedures are described in the supplementary information. With the DSC and TGA results, we defined the end of the first ramp as a dry state where no water is adsorbed in the adsorbents. Experimental data obtained from the DSC and TGA experiments with partially saturated adsorbents are shown in Fig. 2 for 13X and MgY zeolites, and MOF-801. As shown in Fig. 2 (a), the first ramps (ramp 1) have distinctly higher heat flow rates associated with latent heat compared to the second ramps (ramp 2), which were associated with sensible heat. This is expected, since the vapor desorption was carried out during the first ramp, while only the sensible heat of dry adsorbents was responsible for heat flow during the second ramp. Desorption due to heating was observed up to around 350 °C for zeolites and 100 °C for MOF-801, which was observed both with DSC and TGA. The change in mass during the desorption processes (ramp 1) and the second ramp (ramp 2) was monitored with the TGA, as shown in Fig. 2 (b). From the TGA results, the vapor uptake was evaluated with the amount of mass reduced; 13X, MgY and MOF-801 were partially saturated with 31–32, 35–36, and 19–20 wt.% of water vapor, respectively. These uptake measurements were found consistent over multiple runs, as shown in Fig. 1 and Figure S2. The amount of nitrogen adsorbed at these operating conditions was also found to be negligible with the TGA, as shown in Fig. 2 (b). Measurements were repeated for 3 to 5 times in each experimental condition to obtain a 95% confidence interval from the standard error method^29^,^30^.

### Characterization of integral enthalpy of adsorption

We used thermodynamic analysis with the DSC and TGA measurements to determine the integral enthalpy of adsorption. In the analysis, it was assumed that the desorption kinetics in both DSC and TGA experiments are identical, assuming intra-crystalline vapor transport characteristics within adsorbent crystals in the DSC and TGA experiments are identical, and negligible pressure drop across the DSC pans due to purging flow rate. This assumption is further justified in the supplementary information (S.4 and Figure S3). A thermodynamic analysis was carried out using the DSC crucibles as the control volume (CV) and applying the simplified 1^st^ Law of Thermodynamics for an open system (see supplementary information for details), given by


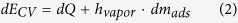


Only the heat transfer interaction, 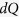, monitored between the CV and DSC, and the vapor enthalpy flow are shown in Eqn (2), where the change in adsorbed phase mass, 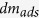, was monitored with the TGA. 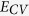 and 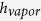 are the total energy within the CV and vapor enthalpy, respectively.

**Figure 3:**
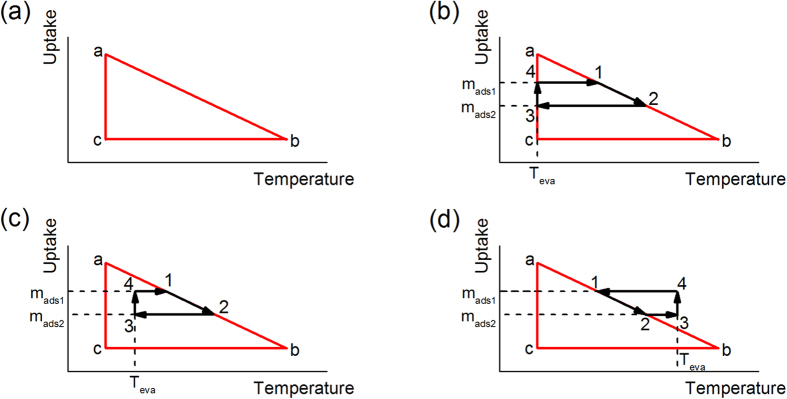
Thermodynamic cycle plotted in uptake vs. temperature. (**a**) Thermodynamic cycle representing an adsorbent undergoing desorption (process a to b), cooling down (process b to c), adsorption (process c to a) processes between temperatures 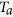 and 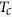. (**b**) to (**d**): Subcycles within the cycle shown in (**a**) with various evaluation temperatures, 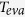. Path 1 to 2 (desorption) is carried out with DSC and TGA experiments.

The overall integral enthalpy of adsorption is calculated by constructing a simple thermodynamic cycle, where an adsorbent undergoes desorption (process a-b), cool-down (process b-c), and adsorption (process c-a), as represented in Fig. 3 (a). In this approach, we consider only the heat transfer and the enthalpy flow between the environment and the CV. Processes a-b and b-c were characterized experimentally with the DSC and TGA, and process c-a is obtained by applying the 1^st^ Law to the entire thermodynamic cycle a-b-c-a. The integral form of Eqn (2) for the desorption process, a-b, is


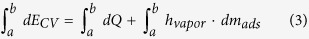


where both the heat transfer interaction and the change in adsorbed phase mass were monitored with the DSC and TGA, respectively. Similarly for the cool-down process,


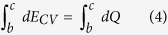


Heat transfer during the process is monitored with the DSC ramp 2. The 1^st^ Law for the entire cycle is,


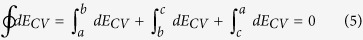


where the total change in energy during the cycle should sum to zero by definition of the 1^st^ Law. Combining Eqns (2) through (5) and rearranging for process c-a, the change in the energy within the CV during the physisorption process is


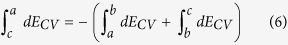


**Figure 4:**
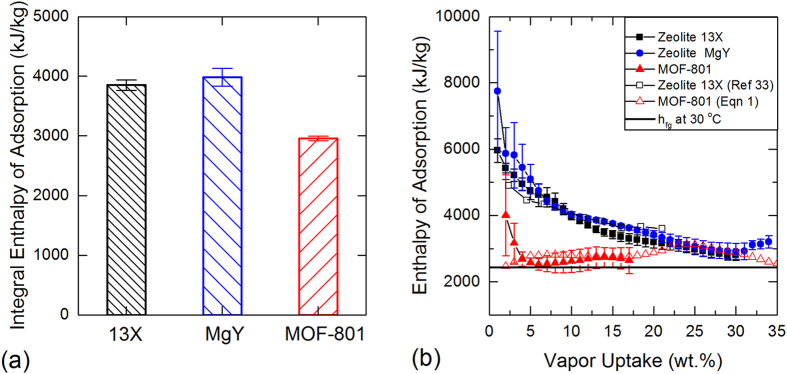
Characterized enthalpies of adsorption. (a) integral adsorption enthalpies and (b) adsorption enthalpies as function of vapor uptake, using Eqns (7) and (17), for 13X and MgY zeolites, and MOF-801 with water pairs at 30 oC. Integral enthalpies are averaged over 31-32 wt.%, 35-36 wt.%, and 19-20 wt.% vapor uptakes for 13X and MgY zeolites, and MOF-801, respectively. Errors reported herein are 95% confidence interval estimated from calculated adsorption enthalpies from all measurements^29^,^30^. Previous calorimetric study^33^of 13X and isosteric enthalpy of MOF-801 from Fig 1(b) are also shown. (Latent heat of evaporation of water at 30 °C, h_fg_ = 2430 kJ/kg).

The change in internal energy of the CV during the isothermal process c-a is composed of both adsorbent and adsorbed vapor. However, the change in energy for the inert adsorbent is zero during the constant temperature process. For an isobaric process, 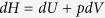, where 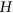 and 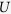 are the enthalpy and internal energy, respectively. If the enthalpy of adsorption is on the same order as the latent heat of vaporization and the specific volume of the adsorbed vapor is on the same order as the liquid water, the specific integral enthalpy of adsorption, 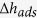, at the initial temperature for the DSC and TGA experiments is then


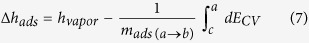


where 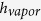 is the enthalpy of the vapor at room temperature (initial temperature of the DSC and TGA experiments) and 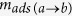 is the amount of the vapor desorbed during the process a–b. 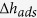 calculated using Eqn (7) for zeolite 13X-water pair was 3852 ± 87 kJ/kg_water_, averaged over 31–32 wt.% vapor uptake. For MgY zeolite and MOF-801, the values were 3985 ± 150 kJ/kg_water_ (averaged over 35–36wt.% vapor uptake) and 2960 ± 39 kJ/kg_water_ (averaged over 19–20wt.% vapor uptake), respectively. Errors reported herein are 95% confidence interval based on the standard error of characterized specific integral enthalpies of adsorption from the two different ramp rates. These values are equivalent to the average energy densities for the given adsorbent-adsorbate system, presented in Fig. 4 (a), which agrees well with direct calorimetric measurements (supplementary information).

### Enthalpy of adsorption as function of uptake

We continue this analysis to estimate the enthalpy of adsorption as a function of uptake. Integration of Eqn (2) between two temperatures, 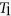 and 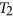, for the desorption process, we obtain the following,


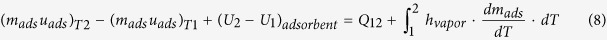


where 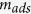 is the mass of the adsorbed vapor and 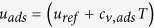 is the internal energy of the adsorbed vapor, 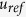 is the internal energy at the reference state, and 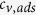 is the specific heat of the adsorbed vapor at constant volume. The change in the internal energy of the solid adsorbent, 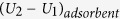, is represented in Eqn (4). If a linear temperature dependence of the internal energy of the adsorbed vapor is assumed (or if the internal energy is nearly constant) between *T*_*1*_ and *T*_*2*_, Eqn (8) can be represented as


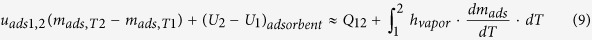


with the accuracy of this equation improving with smaller temperature differences. The specific internal energy of the adsorbed vapor, 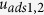, between 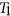 and 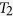 is a both temperature- and uptake-dependent property, and the enthalpy change from the adsorbed vapor to the vapor state is then


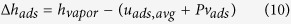


If the specific volume of the adsorbed vapor, 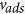, is assumed to be the liquid water and 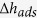 is assumed to be on the same order as the latent heat of vaporization. Then *P*ν_*ads*_ is approximately 10^−5^ of 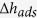. With 0.01% uncertainty, we can express a simplified Eqn (10):


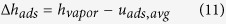


where 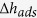 is both the temperature- and uptake-dependent property

To estimate 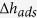 as a function of the uptake at constant temperatures, the thermodynamic cycle shown in Fig. 3 (a) is represented with smaller cycles, as shown in Fig. 3 (b–d), by choosing a temperature interval, 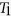 and 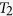. Performing energy balances around the represented cycle and in the process 1 to 2, applying the 1^st^ Law, gives


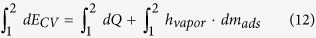


For the processes 2 to 3 and 4 to 1, we have,


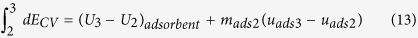



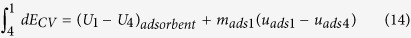


where the changes in the internal energy of the solid adsorbent, 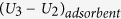 and 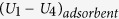, are known from the DSC measurements during ramp 2, equivalent to the sensible heat transfer. The mass of the adsorbed vapor within the CV is 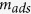. The changes in the internal energy of adsorbed vapor during the processes 2 to 3 and 4 to 1, 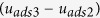 and 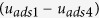, are unknowns. For the process 3 to 4, an adsorption process, we have the 1^st^ Law as shown in Eqn (15),


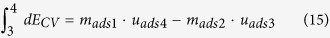


if the linear behavior of the internal energy of the adsorbed vapor between states 3 to 4 is assumed, Eqn (15) can be simplified as


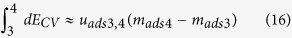


where 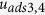 is the specific integral energy of adsorbed vapor between states 3 and 4, evaluated at the constant temperature, 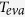. Since the net change in internal energy during a cycle is zero, by combining Eqns from (12) to (16), 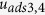 is


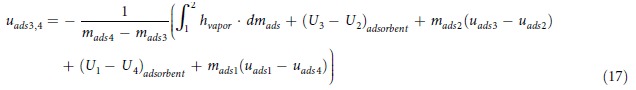


In Eqn (17), the only unknown parameter on the right-hand side is the internal energy of the adsorbed vapor. However, if the internal energy of the adsorbed vapor is assumed to match the saturated liquid water^31^,^32^, we can estimate the enthalpy of adsorption as a function of the uptake, using Eqn (17) as shown in Fig. 4(b) for 13X and MgY zeolites, and MOF-801 at 30 °C. The good agreement between the characterized enthalpy of adsorption for zeolite 13X with the previous direct calorimetric study^33^ justifies this assumption. The temperature intervals used for the calculations are 15 °C for zeolites and 5 °C for MOF-801.

**Figure 5:**
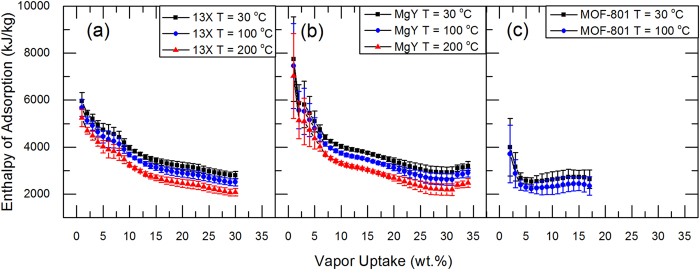
Enthalpies of adsorption as function of vapor uptake at various temperatures for (a) 13X and (b) MgY zeolites, and (c) MOF-801 calculated using model present in Fig. 3. Errors reported herein are 95% confidence interval estimated from calculated adsorption enthalpies from all measurements^29^,^30^.

With the described model, estimating the enthalpy of adsorption at various temperatures by varying the evaluation temperature, 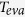, is possible, as shown in Fig. 3. Adsorption enthalpies evaluated at various temperatures for 13X and MgY zeolites, and MOF-801 are also obtained, as shown in Fig. 5.

## Discussions

The specific integral enthalpies of adsorption with vapor for 13X and MgY zeolites, and MOF-801 were estimated by the proposed technique, as shown in Fig. 4(a). As expected, type I zeolites have a higher average enthalpy of adsorption compared to MOF-801. Zeolites have a steep increase in the adsorption energy, greater than twice the latent heat, near the low uptake region, below 5wt.%. In contrast, MOF-801 has a nearly constant enthalpy of adsorption over a wide range of vapor uptakes, as evident in Fig. 4(b). For comparison, the isosteric enthalpy of adsorption for a MOF-801 and water pair, as shown in Fig. 1 (b), is overlaid in the same figure, showing good agreement with the results obtained by our approach.

One of the assumptions to calculate the enthalpy of adsorption using Eqn (17) is the internal energy of the adsorbed vapor being equal to the saturated liquid water. Sensitivity analysis was carried out by varying the internal energy of adsorbed vapor from being equal to the saturated liquid water and saturated vapor. This was performed by varying the adsorbed vapor-specific heat at constant volume, 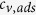, as 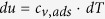. The ice phase has a specific heat between the liquid phase and the vapor phase; therefore, this analysis provides the bounds covering all three phases and is plotted in Figure S4 (supplementary information). Variations in adsorption enthalpies by choosing different internal energies is 10–20% for zeolites and minor for MOF-801 (Figure S4) in the initial loading regime, this is in fact, our model uses the difference in internal energies between two states, the state at the evaluation temperature and the state at the actual desorption temperature. Internal energy of saturated vapor decreases beyond about 200 °C while the internal energy of liquid water continues to rise. Therefore, this variation in adsorption enthalpies becomes larger for adsorbents heated up to higher temperatures. For the purpose of characterizing the enthalpy of adsorption as a function of the uptake accurately, characterizing the specific heat/internal energy of the adsorbed vapor indeed requires using our proposed approach. However, estimating the adsorption energy as a function of the uptake and temperature is also possible with reasonable accuracy using this approach. Likewise, calculating the overall specific integral enthalpy of adsorption using Eqn (7) does not require knowledge of the specific heat, as discussed in this paper, which also matches well with the direct calorimetric measurements (supplementary information).

With the present model, the adsorption enthalpies as a function of the vapor uptake at various temperatures were evaluated, as shown in Fig. 5. Temperatures of 30 °C, 100 °C, and 200 °C were used for zeolites, and 30 °C and 100 °C for MOF-801. Note that it is not necessary for these adsorbents to have the same vapor uptakes at the elevated temperatures. The calculated enthalpy variations due to the temperature elevations are based on the variations observed from the latent heat as a function of temperature. By preparing the partially saturated adsorbent samples with higher vapor uptakes, characterizing the adsorption enthalpies in a wider range of uptake is possible. Higher resolution and accuracy of the proposed technique can also be achieved using a DSC-TGA combined instrument. As water adsorption promises to become an important field of scientific research, the thermodynamic model and method presented in this work will serve as an important technique to characterize one of the most essential properties, enthalpy of adsorption, of various adsorbent-adsorbate systems.

## Additional Information

**How to cite this article**: Kim, H. *et al.* Characterization of Adsorption Enthalpy of Novel Water-Stable Zeolites and Metal-Organic Frameworks. *Sci. Rep.* **6**, 19097; doi: 10.1038/srep19097 (2016).

## Supplementary Material

Supplementary Information
