# Supplementary material for: Characterization of Adsorption Enthalpy of Novel Water-Stable Zeolites and Metal-Organic Frameworks
Source: Sci Rep. 2016 Jan 22;6:19097. doi: 10.1038/srep19097 (PMC4726357; doi:10.1038/srep19097)
Supplement: Supplementary Information [file srep19097-s1.doc]

**SUPPLEMENTARY INFORMATION**

**Characterization of Adsorption Enthalpy of Novel Water-Stable Zeolites and Metal-Organic-Frameworks**

Hyunho Kim,^1^ H. Jeremy Cho,^1^ Shankar Narayanan,^1^ Sungwoo Yang,^1^ Hiroyasu Furukawa,^2^ Scott Schiffres,^1^ Xiansen Li,^1^ Yue-Biao Zhang,^2^ Juncong Jiang,^2^ Omar M. Yaghi,^2^ and Evelyn N. Wang^1^*

^1^ Department of Mechanical Engineering, Massachusetts Institute of Technology, 77 Massachusetts Ave, Cambridge, MA 02139, United States

^2^ Department of Chemistry, University of California – Berkeley, Materials Sciences Division, Lawrence Berkeley National Laboratory, and Kavli Energy NanoSciences Institute at Berkeley, Berkeley, California 94720, United States

**Corresponding Author**

*Email: enwang@mit.edu

**S.1 Sample Preparation and Experimental Procedures**

MgY and 13X zeolites were stored in an as-received container under atmospheric conditions, which should not affect their adsorption characteristics,[^1^](#_ENREF_1) and MOF-801 was stored under an inert condition with nitrogen in a glove box system. Adsorbent powders were first dehydrated for 2-3 hours. In this regard, zeolite samples were dehydrated at 400 ^o^C in an oven, and MOF-801 samples were dehydrated at 70 ^o^C in a gravimetric vapor sorption analyzer (Q5000SA, TA Instruments) with dry nitrogen purging gas flow of 200 ml/min until the sample weight stabilized. These drying processes were followed by sample exposure to 60 % relative humidity at 25 ^o^C, using a mixture of deionized water vapor and nitrogen gas in the sorption analyzer at atmospheric pressure to saturate the samples with vapor at the given condition. Dehydrated high temperature zeolite samples were transferred directly from the oven to the sorption analyzer with the sample pan prior to the saturation process. After saturation, 5 to 10 mg of the saturated samples were transferred within a glass container under atmospheric conditions and tested in a DSC (Discovery DSC, TA Instruments) and a TGA (Discovery TGA, TA Instruments) with identical experimental conditions. Sample exposure duration to the atmospheric environment was roughly 10-15 minutes. The consistency observed in the uptake measurements of all adsorbents is shown in Figure 1 and Figure S2, supports that the exposure duration did not affect the adsorption equilibrium significantly. In addition, MOF-801 was tested with the dynamic vapor sorption analyzer (DVS) after exposure to the atmospheric condition for ~1 hour, and no noticeable degradation was observed. DSC- and TGA-tested zeolites were also tested using the DVS, and degradation, from high temperature drying and testing conditions, was likewise not observed.

The DSC and TGA tests were carried out with two temperature ramps by cooling samples down to the initial temperature after ramp 1. Zeolite samples were ramped to 500 ^o^C at 1 and 3 ^o^C/min and MOF-801 samples were ramped to 115 ^o^C at 0.5 and 1 ^o^C/min from room temperature while maintaining nitrogen purging gas flow at 25 ml/min. Artificial start-up effects during the initial heating in the DSC experiments were found to be negligible with the ramp rates used in this study.[^2^](#_ENREF_2) To maintain similar conditions in the DSC and TGA experiments, the same DSC sample pan and lid (Tzero hermetic pan and lid, TA Instruments) were used in the TGA, with a pin hole in the lid to facilitate vapor removal during the desorption, as illustrated in Figure S1.

**S.2 Simplification of the 1^st^ Law**

The 1^st^ Law of Thermodynamics for an open system with a single heat transfer interaction, $dQ$, is

|  | $dE_{CV}=dQ-\left(dW_{rev}-TdS_{gen}\right)+\sum_{i,in} h_{i}\cdot dm_{i}-\sum_{j,out} h_{j}\cdot dm_{j}$ | (S1) |
| --- | --- | --- |

where $E_{CV}$, $Q$, $W_{rev}$, $T$, $S_{gen}$, $h$, and $m$ are the change in total energy, heat flow rate monitored with the DSC, reversible work transfer, temperature, generated entropy, enthalpy and mass within the control volume, respectively. For simplicity, the contributions from potential and kinetic energies were neglected in Eqn (S1). Reversible work transfer can be represented with a simple form $pdV$ ($p$ and $V$ are pressure and volume, respectively), which takes into account the change of mass in the CV, and was found to be relatively small compared to the change in enthalpy. If the specific volume of the adsorbed vapor is assumed to match liquid water, then $pdV$ is found to be 10^-4^ times the enthalpy flow shown in Eqn (S1). The enthalpy flow of nitrogen purging gas can be neglected since nitrogen was not adsorbed, and the vapor uptake was significantly larger.

The entropy generation term in Eqn (S1) is a result of combining the 2^nd^ Law of Thermodynamics with the 1^st^ Law. The generation of entropy is expected to be mainly due to the temperature gradient within the powdered adsorbent[^3^](#_ENREF_3) and the gaseous mixing, as these processes are irreversible. The entropy generation during a conduction heat transfer process, $S_{gen,cond}$, through a finite thickness and thermal conductivity with a constant heat flow, $q$, across a temperature difference $T_{2}$ to $T_{1}$ is given by[^3^](#_ENREF_3)

|  | $S_{gen,cond}=q\left(\frac{1}{T_{1}}-\frac{1}{T_{2}}\right)$ | (S2) |
| --- | --- | --- |

The heating rates used for the experiments were 0.5-3 ^o^C/min and the thermal conductivity of the adsorbent samples was approximately 0.1 W/mK. Using a natural convection heat transfer coefficient of 10 W/m^2^K,[^6^](#_ENREF_6) the Biot number (*Bi = hL/k*, where *h*, *L* and *k* are the convective heat transfer coefficient [*W/m^2^K*], characteristic length scale [*m*] and thermal conductivity [*W/mK*], respectively) was found to be on the order of 10^-1^. Additionally, based on the Fourier number (*Fo = αt/L^2^*, where *α* and *t* are the thermal diffusivity [*m^2^/sec*] and time [*sec*], respectively) of 3/2, the characteristic time scale was found to be ~ 1 sec. Therefore, the system was approximated as a lumped capacitance with negligible temperature gradients.

The entropy generation from the gaseous mixing was also scaled with an assumption of the ideal gas behavior in the gaseous phase and applying the ideal gas entropy of mixing, given by

|  | $dS_{mix}=-k_{B}\sum_{i} \ln\left(\frac{N_{i}}{N}\right)dN_{i}$ | (S3) |
| --- | --- | --- |

where $S_{mix}$, $k_{B}$, and $N$ are the entropy of mixing, the Boltzmann constant, and the number of molecules, respectively. The scaling assumed the following: (1) the initial mole fraction of the vapor within the CV being 0.02 and eventually going down to zero with a temperature ramp from 25 ^o^C to 200 ^o^C, (2) the mass of the adsorbed phase being 1 g, and (3) the CV being 1 L. With these assumptions, $TdS_{mix}$ was found to be an order of 10^-5^ of the enthalpy flow term in Eqn (S1), allowing us to neglect the generation of entropy during the experiments. Consequently, the 1^st^ Law simplified to,

|  | $dE\approx dQ+h_{vapor}\cdot dm_{ads}$ | (S4) |
| --- | --- | --- |

Only the heat flow rate, $dQ$, monitored with the DSC, and the vapor enthalpy flow are shown in Eqn (S4), where the rate of vapor desorption, $dm_{ads}$, was monitored with the TGA.

**S.3 Direct calorimetric measurements of 13X and MgY zeolites**

To validate the overall enthalpies of adsorption calculated using Eqn (7), direct calorimetric measurements of 13X and MgY zeolites were carried out using a method as reported. Samples (25*25*2 mm) were fabricated with known dehydrated weights with thermally conductive copper foams (100 ppi copper foam, Shanghai Winfay Metal & Plastic Manufacturing Co., Ltd) by immersing the foam in a liquid water and zeolite mixture for ~ 5 hours. Fabricated samples were interfaced with a heat flux sensor (HFS-4, OMEGA engineering) with a thermally conductive pad (A15896-02, Laird Technologies) inside a vacuum environmental chamber system.[^9^](#_ENREF_9) Samples were dehydrated at high vacuum (~ 1 Pa) at temperature around 110 ^o^C prior to the measurements. After the desorption process, degassed vapor was introduced inside the chamber maintaining the chamber pressure around 2500 Pa with the sample temperatures maintaining around 22 ^o^C using a base plate interfaced with coolant lines. Relative pressure ($P/P_{sat}$ [Pa/Pa], absolute pressure over saturation pressure) during the measurements was roughly 94% by knowing the pressure inside the chamber and the sample temperature. The enthalpy of adsorption was calculated by applying the first law around the sample, given by

|  | $\Delta h_{ads}=-\frac{Q_{HFS}}{m_{ads}}$ | (S5) |
| --- | --- | --- |

where $Q_{HFS}$ is the total thermal energy [kJ] measured by the heat flux sensor during the experiments and is negative value by the direction of heat transfer from the sample to the sensor. Amount of adsorbed vapor, $m_{ads}$[kg], was estimated with the relative pressure at the end of the heat flux measurements using the adsorption isotherms shown in Figure 1. Heat losses during the experiments were found to be negligible by comparing the conduction and convection thermal resistances. Assuming the thermal conductivity of the samples were on the order of 1 W/mK[^10^](#_ENREF_10) and the convection heat transfer coefficient to be on the order of 10 W/m^2^K,[^6^](#_ENREF_6) thermal resistance ratio of the conduction and convection heat transfers are on the order of 10^-2^, allowing us to neglect the convective losses during the experiments. Directly measured overall enthalpies of adsorption for 13X and MgY zeolites were 3808 ± 398 kJ/kg_water_ (averaged over 32-34wt.% vapor uptake) and 4041 ± 452 kJ/kg_water_ (averaged over 38-40wt.% vapor uptake), respectively. These measurements agree well with the indirect DSC and TGA measurements.

**S.4 Assumption of identical kinetics in DSC and TGA experiments**

One of the most important assumptions made to use the proposed technique is the assumption of identical kinetics during the DSC and TGA experiments. First, unpacked powdered adsorbents were used in the experiments in which inter-crystalline vapor transport between adsorbent crystals is not a limiting mechanism of mass transfer as intra-crystalline vapor transport within the adsorbent crystals dominates.[^11^](#_ENREF_11) Since intra-crystalline vapor transport is a material characteristic, the behavior should be the same whether a DSC or TGA experiment is being run. Second, the pressure variation on the pin holes due to purging gas flow in the DSC and TGA chambers is negligible. Using the Bernoulli equation (which would provide a theoretical maximum stagnation pressure), a pressure increase of less than 0.005 Pa for the TGA case was estimated. Given that the mass and heat transfer mechanisms are similar and two different experiments were subjected to the same boundary conditions, the identical kinetics assumption is reasonable. This was further validated with a number of runs made with 13X zeolite where samples subjected to different ramp rates had noticeably small errors in the calculation of adsorption enthalpies as a function of vapor uptake, as shown in Figure S3. We believe larger variations observed from experiments made with MgY zeolite and MOF-801 were due to variations in the materials. For instance, MgY zeolite requires an additional ion exchange process from commercial NaY zeolite[^1^](#_ENREF_1) and MOF-801 is synthesized only in small quantities (1-10 g).[^12^](#_ENREF_12) Only zeolite 13X samples had highly consistent adsorption isotherm measurements as MOF-801 and MgY zeolite are both new classes of materials recently developed. Errors reported in Figure 4 is a 95 % confidence interval estimated from the standard error. Measurements were repeated 3 to 5 times and measurement errors were estimated via propagation of uncertainty to be smaller than the standard errors with the assumption of ideal kinetics during the DSC and TGA experiments.

**References**

1 Li, X. *et al.* Zeolite Y adsorbents with high vapor uptake capacity and robust cycling stability for potential applications in advanced adsorption heat pumps. *Microporous and Mesoporous Materials* **201**, 151-159 (2015).

2 Thomas, L. C. Interpreting Unexpected Events and Transitions in DSC Results.

3 Bejan, A. Second law analysis in heat transfer. *Energy* **5**, 720-732 (1980).

4 Jakubinek, M. B., Zhan, B.-Z. & White, M. A. Temperature-dependent thermal conductivity of powdered zeolite NaX. *Microporous and Mesoporous Materials* **103**, 108-112 (2007).

5 Liu, D. *et al.* MOF-5 composites exhibiting improved thermal conductivity. *international journal of hydrogen energy* **37**, 6109-6117 (2012).

6 Mills, A. F. *Heat Transfer*. (Prentice Hall, 1999).

7 Sircar, S., Mohr, R., Ristic, C. & Rao, M. Isosteric heat of adsorption: theory and experiment. *The Journal of Physical Chemistry B* **103**, 6539-6546 (1999).

8 Rouquerol, J., Rouquerol, F., Llewellyn, P., Maurin, G. & Sing, K. S. *Adsorption by powders and porous solids: principles, methodology and applications*. (Academic press, 2013).

9 Miljkovic, N. *et al.* Jumping-droplet-enhanced condensation on scalable superhydrophobic nanostructured surfaces. *Nano letters* **13**, 179-187 (2012).

10 Ashby, M. F. *et al.* *Metal Foams: A Design Guide: A Design Guide*. (Elsevier, 2000).

11 Narayanan, S., Yang, S., Kim, H. & Wang, E. N. Optimization of adsorption processes for climate control and thermal energy storage. *International Journal of Heat and Mass Transfer* **77**, 288-300 (2014).

12 Furukawa, H. *et al.* Water adsorption in porous metal–organic frameworks and related materials. *Journal of the American Chemical Society* **136**, 4369-4381 (2014).

13 Bragg, G. M. *Principles of experimentation and measurement*. (Prentice-Hall Englewood Cliffs, NJ, 1974).

14 Barry, B. A. *Errors in practical measurement in science, engineering, and technology*. (John Wiley & Sons Inc, 1978).


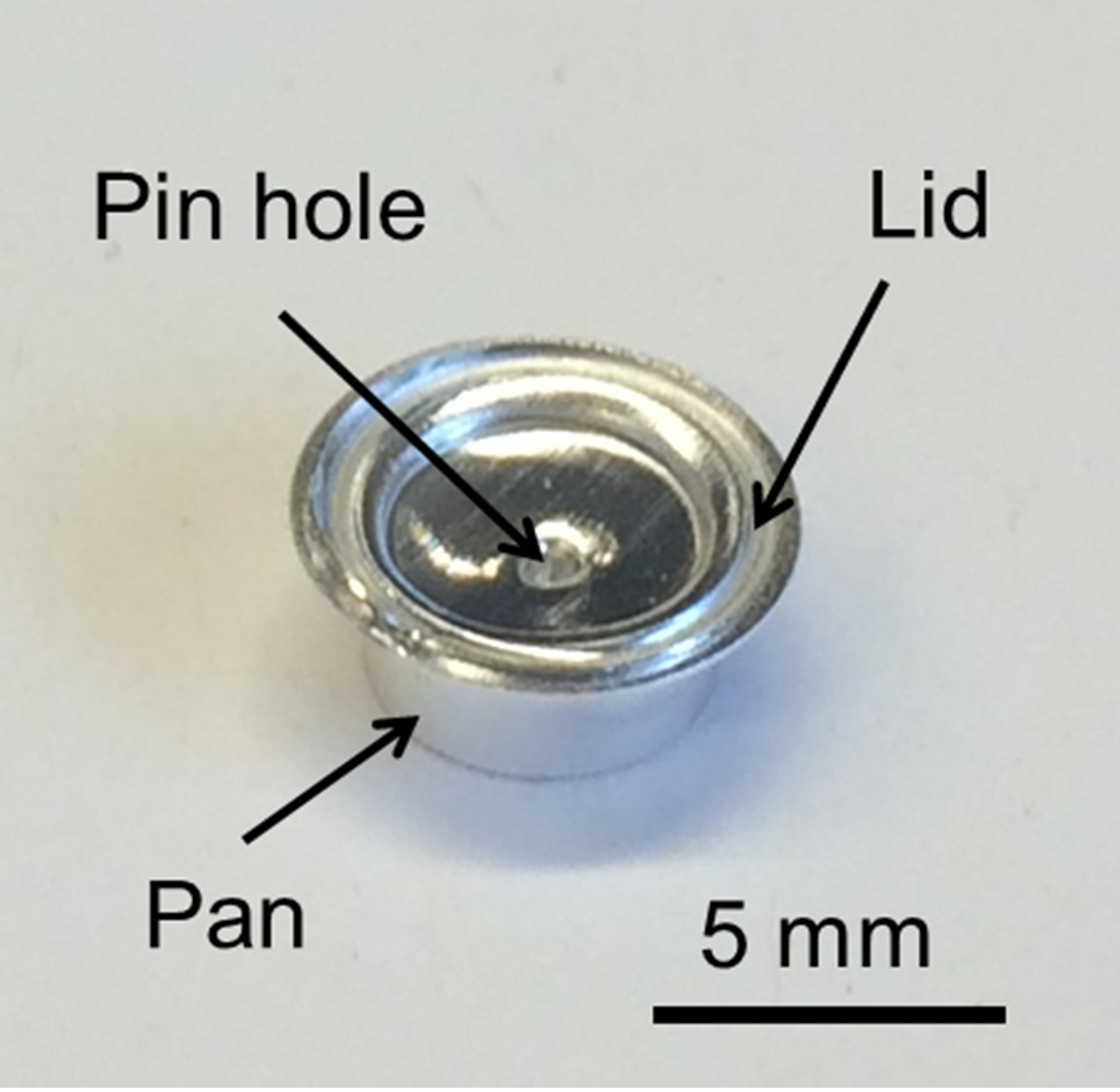


Figure S1. Image of DSC pan and lid. (Tzero hermetic pan and lid, TA Instruments) with a pin hole for vapor removal during DSC and TGA experiments.


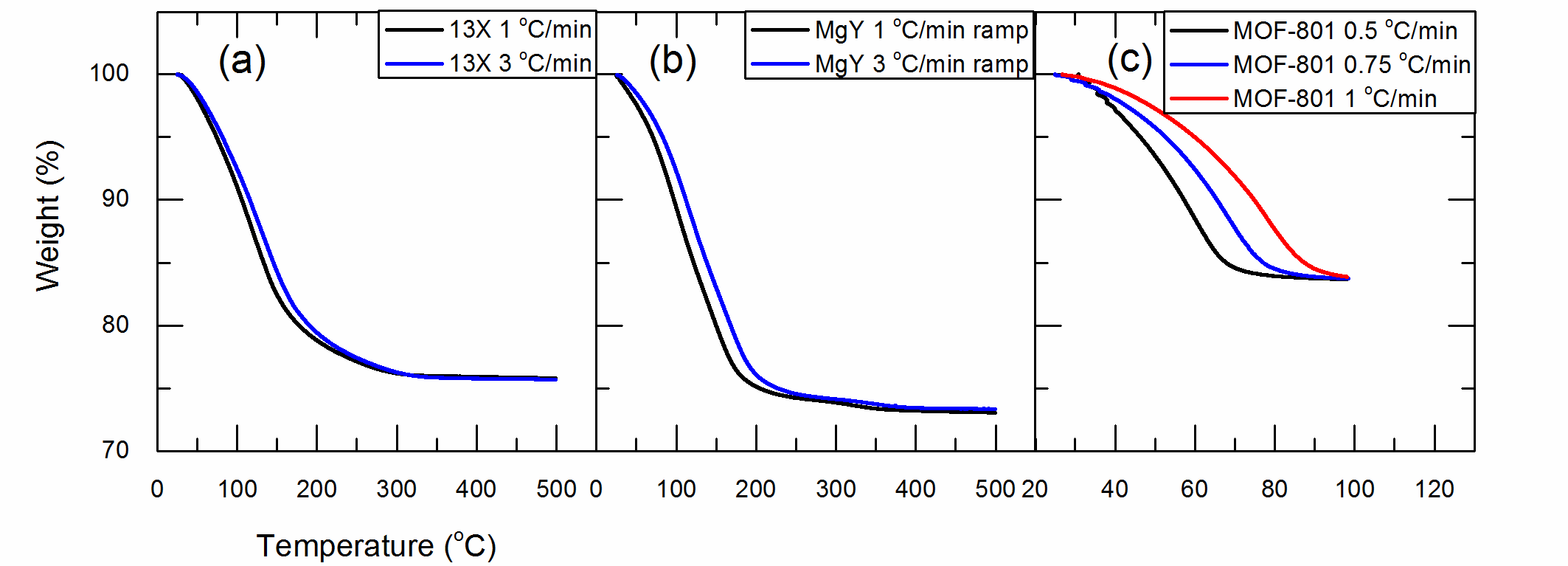


Figure S2. TGA results of 13X and MgY zeolites, and MOF-801 at various ramp rates. Only the first ramp is shown.


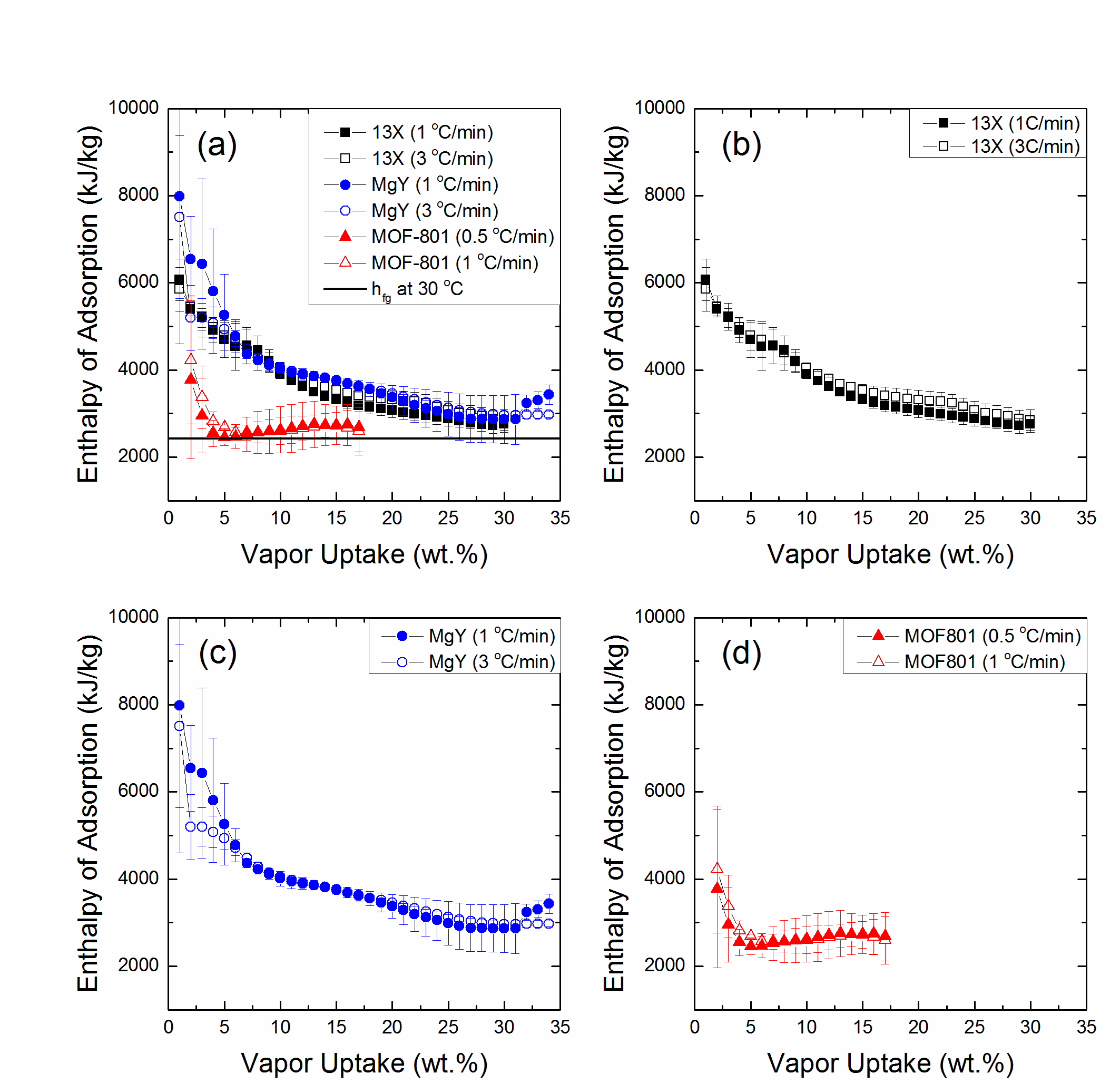


Figure S3. Enthalpy of adsorption as function of uptake at various ramp rates calculated using Eqns (9) and (11) for 13X and MgY zeolites, and MOF-801 at 30 ^o^C.


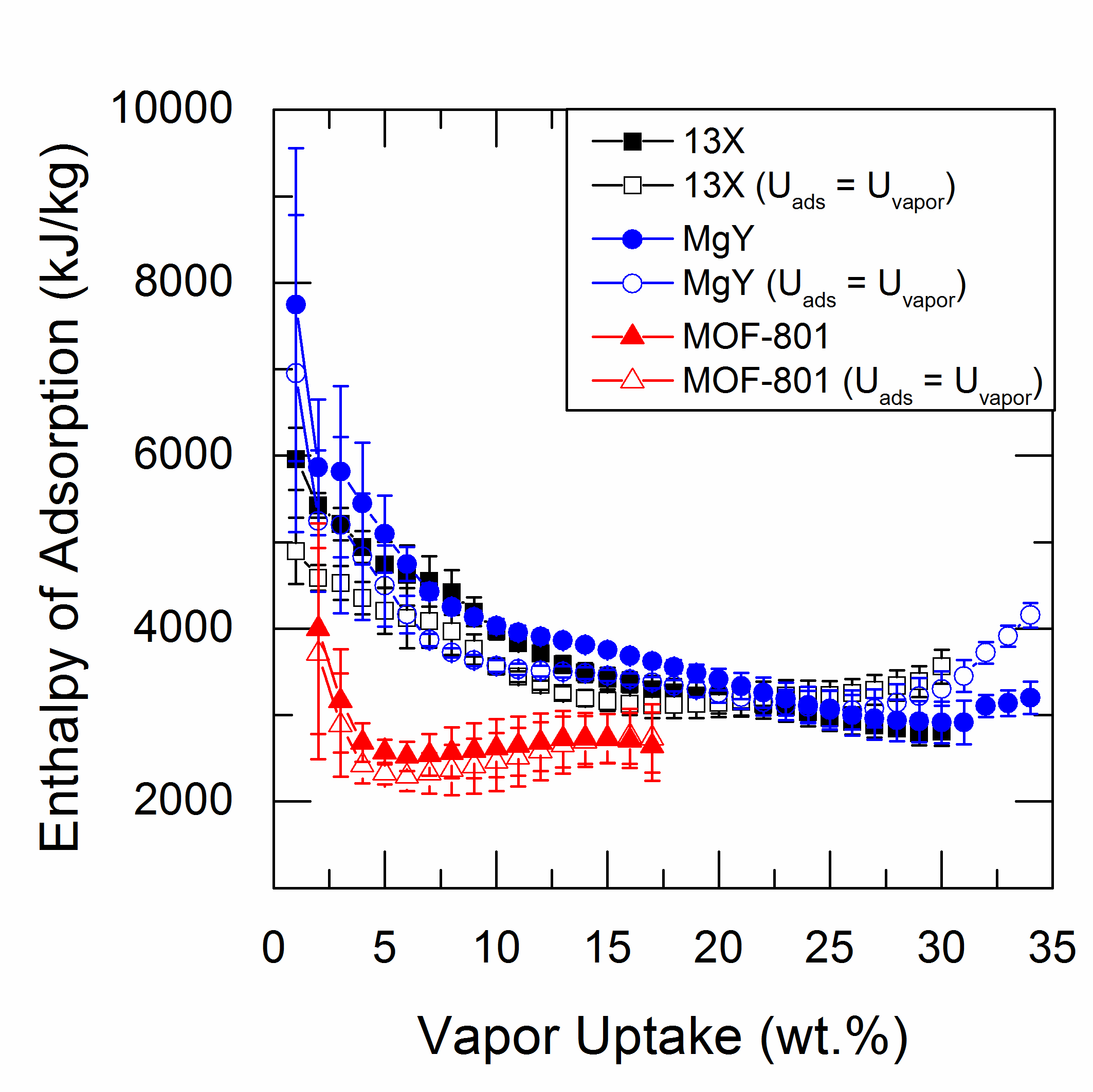


Figure S4. Enthalpy of adsorption as function of uptake calculated using Eqn (17) by varying internal energy of adsorbed vapor at 30 ^o^C. Closed and open data points were calculated with temperature-dependent saturated liquid water and saturated vapor internal energies, respectively. Errors reported herein are 95 % confidence interval estimated from calculated adsorption enthalpies from all measurements.
